# Adrenal function, morbidity and mortality in patients with COVID-19

**DOI:** 10.1007/s12020-025-04401-w

**Published:** 2025-09-01

**Authors:** Karina Rozenfeld, Elad Shemesh, Elena Izkhakov, Miguel M Moshiashvili, Eugene Feigin, Ilana Goldiner, Ben-Zion Katz, Karen Tordjman, Gabi Shefer, Yona Greenman

**Affiliations:** 1 Institute of Endocrinology, Diabetes and Metabolism, Rambam Health Care Campus, Haifa, Israel; 2 Institute of Endocrinology, Diabetes, Metabolism and Hypertension, Tel Aviv-Sourasky Medical Center, 6 Weizmann Street, Tel Aviv, 6423906 Israel; 3 Faculty of Medical & Health Sciences, Tel Aviv University, Tel Aviv, Israel; 4 Clinical Biochemical Laboratory, Tel Aviv-Sourasky Medical Center, Tel Aviv, Israel; 5 Laboratory of Hematology, Tel Aviv-Sourasky Medical Center, Tel Aviv, Israel

**Keywords:** Adrenal function, Free cortisol, Stress response, COVID-19, Critical illness

## Abstract

**Purpose:**

Reports of impaired glucocorticoid response in patients with severe COVID-19, associated with increased mortality, have led to the hypothesis that the beneficial effects of dexamethasone in these patients may be mediated by correction of adrenal insufficiency. Considering the limited reliability of total cortisol (TC) in critically ill patients, we sought to assess the incidence of adrenal insufficiency and its relation to negative outcomes according to dexamethasone treatment, using serum free cortisol (SFC).

**Methods:**

Retrospective analysis of 1,971 COVID-19 patients hospitalized in a major medical center from May 2020 to March 2021. In-hospital and 30-day mortality, and the need for ventilation support, were analyzed in relation to SFC levels.

**Results:**

The final cohort comprised 241 patients, 53% of whom had moderate to severe disease. Adrenal insufficiency was diagnosed in 3.5–5.8% in patients with mild and moderate disease, and in 7% of patients with severe disease. SFC was a strong independent variable for all outcome measures, with an Odds Ratio of 1.933 (1.299–2.877), *p* = 0.001; 2.673 (1.770–4.037), *p* < 0.001; and 1.515 (1.136-2.0), *p* = 0.005, for in-hospital mortality, 30-day mortality and the need for assisted ventilation, respectively. Dexamethasone-treated patients exhibited the same pattern of disease-severity-driven cortisol levels, as seen in the whole cohort.

**Conclusion:**

Adrenal insufficiency is rare and unrelated to the beneficial effects of dexamethasone in COVID-19 patients. SFC is a strong predictor of adverse outcomes, emerging as a superior biomarker for disease severity and prognosis, compared to other clinical and inflammatory parameters.

## Introduction

The clinical course of coronavirus disease 2019 (COVID-19) is highly heterogeneous, ranging from mild or asymptomatic infection to severe manifestations, such as inflammatory storm, acute respiratory distress syndrome, and potentially death. While primarily targeting the respiratory system, COVID-19 also affects multiple extra pulmonary organs, leading to cardiovascular and neurological systems complications, among others [1–3].

The endocrine system is also vulnerable to the severe acute respiratory syndrome coronavirus 2 (SARS-CoV-2). The angiotensin-converting enzyme 2 receptor and the transmembrane serine protease 2, crucial for viral entry, are expressed in various endocrine glands, notably the adrenal and, to a lesser extent, the pituitary [4, 5] Autopsy studies have detected SARS-CoV-2 RNA and proteins in these glands of deceased COVID-19 patients, with nearly half showing signs of adrenal damage including ischemia, hemorrhage, or inflammation [6, 7]. Notably, acute adrenal infarction was observed in 23% of 219 patients hospitalized with severe lung lesions, with bilateral damage in 88% of them [8].

The functional implication of these findings remains unclear. Random total cortisol (TC) levels were lower in critically ill COVID-19 patients, compared to non-infected intensive care unit patients [4]. A higher prevalence of central hypocortisolism was found in patients with moderate to severe COVID-19 (38.5%) compared to those with mild disease (6.8%) [9]. Another study found hypoadrenalism more prevalent in moderate versus mild or asymptomatic cases [10]. Finally, in a cohort of 154 hospitalized patients in Iran, TC levels were significantly lower in patients who died, with an odds ratio of 0.74 for each unit increase in TC levels [11].

In strike contrast, patients with COVID-19 have been shown to mount an appropriate and even exuberant cortisol response to stress, with baseline TC levels associated with increased mortality [12, 13]. A meta-analysis confirmed higher TC levels in patients with severe compared with those with mild-to-moderate disease [14]. These seemingly contradictory results may relate to different criteria used to classify the appropriate cortisol response to stress and stratifying COVID-19 severity [4, 9, 10]. Additionally, since over 90% of serum cortisol is protein-bound, decreases in corticosteroid-binding globulin and albumin during acute illness, leading to low TC levels being misinterpreted as adrenal insufficiency. Therefore, serum free cortisol (SFC) is considered a better indicator of HPA axis function during critical illnesses [15–17].

Glucocorticoid treatment significantly reduces morbidity and mortality in severe COVID-19 [13, 18, 19]. Whether correcting possible concurrent hypoadrenalism mediates the beneficial effects of dexamethasone remains to be established. Despite vaccines and less virulent SARS-CoV-2 variants reducing the global COVID-19 burden, identifying adrenal insufficiency (AI) remains critical for appropriate treatment.

This study assessed the prevalence of AI, its relation to clinical outcomes, and the influence of baseline cortisol levels on the clinical response to dexamethasone treatment in hospitalized COVID-19 patients. Additionally, we explore SFC as a biomarker for disease severity and its potential to predict clinical outcomes.

## Materials and methods

### Study population and design

We conducted a retrospective analysis of clinical data from 1971 hospitalized COVID-19 patients, confirmed by RT-PCR, between May 2020 and March 2021.

Eligibility for inclusion was based on the availability of frozen serum collected for routine interleukin-6 (IL-6) measurements within the first 48 h of hospitalization. Patients with a history of adrenal disease, chronic steroid therapy, or those who received glucocorticoids within 48 h prior to blood sampling were excluded. The study protocol was approved by the Institutional Human Studies Ethical Committee (Approval No. TLV-0082-21), and informed consent was waived due to the retrospective nature of the study.

## Variables

Clinical data were extracted from electronic medical files. Elevated blood pressure was defined as ≥ 140/90 mmHg or a history of hypertension and use of antihypertensive medications. Fever was defined as > 38.3 °C. Desaturation was defined as O2 saturation ≤ 93% in room air. COVID-19 severity was defined according to the National Institutes of Health (NIH) guidelines as Asymptomatic, Mild, Moderate, Severe, or Critical [20].

## Events

Adjudicated events included in-hospital mortality, 30-day mortality, and the need for ventilatory support (high-flow nasal cannula, continuous positive airway pressure (cPAP) or mechanical ventilation).

## Laboratory measurements

Blood samples were drawn within 48 h of admission for IL-6 measurement. Serum samples were processed and frozen at − 80 °C within two hours of collection. Samples were thawed only once, immediately prior to free cortisol analysis, which was performed 6 to 18 months after collection depending on the date of hospitalization. Of these, 150 samples were collected during the early morning hours (7 AM to 10:30 AM), while 91 were taken outside this timeframe. Among the latter group, cortisol measurements were performed in samples from 32 patients with severe to critical illness, 11 with moderate disease, and 48 with mild conditions.

*Cortisol Measurement*: Total and free cortisol concentrations were determined following a 24-hour equilibrium dialysis process as described by Limor et al. [21]. After incubation, total cortisol was measured from the liquid above the filter, while free cortisol was measured from the dialysate. Both total and free cortisol levels were quantified using a COBAS 601 analyzer, using the Elecsys Cortisol II assay (Roche Diagnostics).

*Inflammatory and Biochemical Markers*: IL-6 was measured using a chemiluminescence kit on an IMMULITE 2000 Immunoassay System (SIEMENS, Siemens Healthcare Diagnostics Products Ltd.), with a reference range of 0–5.9 pg/mL. Other biomarkers including wide-range CRP, LDH, ALT, and creatinine were analyzed using ADVIA Chemistry Systems (Siemens Healthcare Diagnostics Inc., Tarrytown, NY, USA). Ferritin was quantified on an ADVIA Centaur^®^ XP analyzer (Siemens Healthcare Diagnostics Inc.).

## Hematology parameters

Prothrombin Time (PT)/INR assay was performed utilizing Innovin as a recombinant human tissue factor (Dade, Siemens, Marburg, Germany). D-dimer was analyzed using an immunoturbidimetric Innovance assay (Siemens), and fibrinogen using the Clauss assay utilizing recombinant thrombin (Siemens), all on CS5100 instruments (Sysmex, Kobe, Japan). CBC was analyzed using DxH800 Beckman Coulter CBC analyzers (Brea, CA, USA).

### Statistical analyses

Categorical variables were summarized as frequency and percentages. Non-normally distributed variables were reported as median and interquartile range (IQR). Associations between SFC levels and other continuous variables were evaluated using the Spearman correlation coefficient. The Mann Whitney test assessed associations between continuous variables, while the Chi-square or Fisher’s exact test assessed associations between categorical variables. Variables significantly associated in univariate analysis were included in multivariable analysis, using logistic regression. Forward method using Wald test as criteria for variables inclusion was applied (criterion for inclusion *P* < 0.05). The area under the receiver-operating curve (ROC) and t Youden index evaluated the discriminatory values of TC, SFC, and inflammatory markers for clinical outcomes. All statistical tests were two sided with *p* < 0.05 considered significant. SPSS software (IBM SPSS Statistics for Windows, Ver. 29) was used for all analyses.

## Results

### Baseline characteristics

**Fig. 1:**
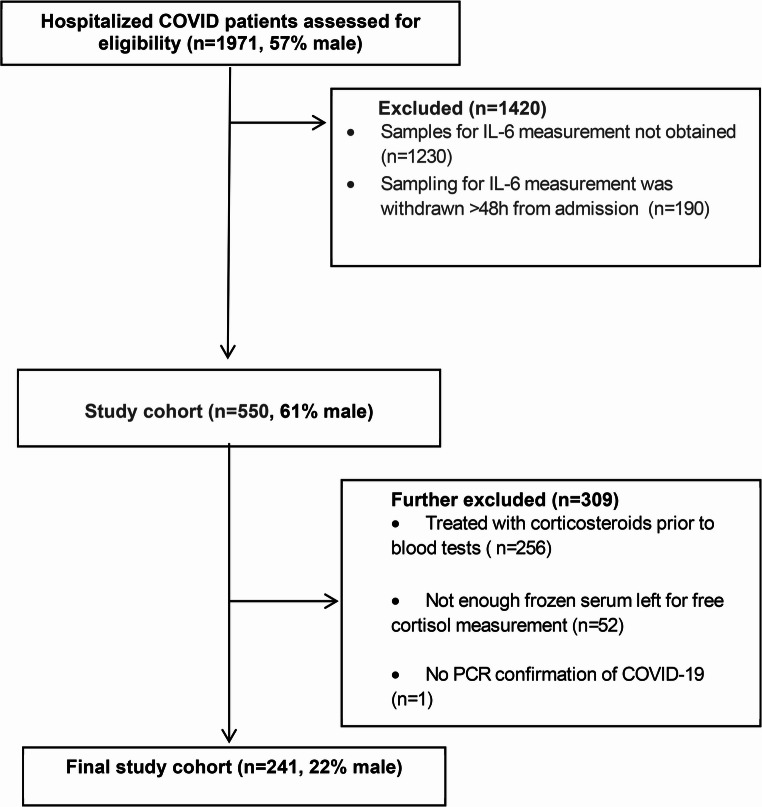
Flow diagram of patients excluded from the initial cohort according to specific criteria

From the initial cohort of 1971 patients (57% men), 241 met the eligibility criteria and were included in the final analysis (Fig. 1). Women represented 78% of the final cohort and were older (70 ± 17 years) than men (57 ± 19, *p* = 0.001). The gender imbalance is attributed to the high rate of male subjects (69.3%) excluded due to prior dexamethasone treatment (Fig. 1). Analysis of this excluded group revealed that 81% had severe disease, which may have required prompt dexamethasone administration. Table 1 outlines the baseline characteristics of the study population. Common underlying conditions included hypertension (46.9%), hyperlipidemia (37.8%), diabetes (30.3%), cardiovascular disease (24.5%) and obesity (23.2%). Most patients presented with fever (69.3%) and respiratory complains (60.2%) and were admitted after a median period of five (2-8) days from the onset of symptoms.

**Table 1:** Baseline characteristics of the whole cohort and as stratified by exposure to dexamethasone

Baseline variables	All patients *N* = 241	Treatment Assignment		
		**Dexamethasone** *N* = 114	**Usual care** *N* = 127	P value
**Age (years)**,** median (IQR)**	69.3 (54.5–82.9)	72.0 (59.2–82.6)	67.3 (51.6–83.7)	NS
**Gender**,** number of males (%)**	53 (22)	14 (12.3)	39 (30.7)	0.001
**Underlying diseases**	N (%)			
Hypertension	113 (46.9)	53 (46.5)	60 (47.2)	NS
Diabetes mellitus	73 (30.3)	39 (34.2)	34 (26.8)	NS
Hyperlipidemia	91 (37.8)	50 (43.9)	41 (32.3)	0.08
Cardiovascular disease	59 (24.5)	28 (24.6)	31 (24.4)	NS
Obesity	56 (23.2)	37 (32.5)	19 (15.0)	0.002
Chronic kidney disease	36 (14.9)	18 (14.2)	18 (15.8)	NS
Hypothyroidism	34 (14.1)	15 (13.2)	19 (15.0)	NS
Heart failure	33 (13.7)	17 (14.9)	16 (12.6)	NS
Respiratory disease	28 (11.6)	18 (15.8)	10 (7.9)	0.07
Smoking	26 (10.8)	12 (9.4)	14 (12.3)	NS
Solid cancer	10 (4.1)	4 (3.5)	6 (4.7)	NS

Upon admission, patients were classified as asymptomatic (positive RT-PCR for SARS-CoV-2 in subjects hospitalized for other reasons, 3.7%), or as having mild (43.2%), moderate (17.4%), severe (33.6%) or critical (2.1%) COVID-19 based on NIH criteria. Due to their low number, asymptomatic subjects were grouped with those with mild disease, and critical patients were grouped with patients with severe disease, creating three groups: mild, moderate, and severe. Further details on signs, symptoms and baseline laboratory values at presentation are in Supplementary Tables 1 and 2.

### Medical treatment

Overall, 114 (46.8%) patients were treated with dexamethasone (DT), 81 (71%) of whom had severe or critical disease. Eleven patients with mild and 20 with moderate disease at presentation deteriorated clinically during the hospitalization and were subsequently treated with DT. A higher percentage of women (30.7%) compared to men (12.3%, *p* = 0.001) received DT, likely reflecting the high number of men excluded from the study due to prior DT administration. Obesity was more prevalent in DT-treated patients than in the supportive group (32.5% vs. 15%, *p* = 0.002), but no significant differences were noted in age, or other comorbidities (Table 1).

### Patients with possible adrenal insufficiency

Baseline TC levels below 3 µg/dl [22] or below 5 µg/dl [23] are considered indicative of adrenal insufficiency, without the need for additional stimulatory testing. New thresholds indicating a normal TC response to ACTH stimulation test were established for the Roche Elecsys Cortisol II assay, ranging from 12.7 µg/dl [24] to 14.6 µg/dl [25]. Random TC levels below 10 µg/dl are used to diagnose critical illness-related corticosteroid insufficiency (CIRCI) [26]. Finally, a SFC level over 0.91 µg/dl was the cut-off indicating a normal response to the 1 µg ACTH stimulation test, using the previous Roche assay [21], corresponding to 0.73 µg/dl using the current assay.

The diagnosis of possible adrenal insufficiency based on basal cortisol levels was considered in patients with mild and moderate disease, in whom blood samples were drawn during early morning hours. Among patients with mild (*n* = 65) and moderate (*n* = 32) disease, none had TC levels below 5–3 µg/dl, and three had levels below 10 µg/dl. None of these patients received DT, and there were no in-hospital or 30-day fatalities in this group. Given the high variability in clinical presentation (e.g. presence or absence of fever), patients with cortisol levels below the normal ACTH-stimulation test threshold were identified. Admittedly, TC levels below this threshold may have been adequate for a particular patient stress level. Twelve mild disease patients had TC levels below 14.6 µg/dl, nine of whom also failed the 12.7 µg/dl cut-off. One moderate disease patient had a cortisol value below 12.7. None received DT, and no in-hospital or 30-days deaths were recorded.

In patients with severe and critical disease, all samples were analyzed, as these subjects would be expected to mount an adequate response to stress irrespective of blood sample timing. Among 86 patients with severe to critical disease, five had TC below 14.6 µg/dl, of whom four had SFC below 0.73 µg/dl. There were six patients with severe disease and random SFC levels below 0.73 µg/dl, two of whom had TC levels above14.6 µg/dl. All received DT, and none died in hospital or within 30 days.

Among the 21 in-hospital deaths (18 with severe disease), none had random TC < 14.6 µg/dl or SFC < 0.73 µg/dl. Among 20 patients who died post-discharge and within 30 days, none had TC below 14.6 µg/dl, and one had a borderline SFC value of 0.735 µg/dl; this patient received DT during hospitalization.

### Dexamethasone-treated patients

**Fig. 2:**
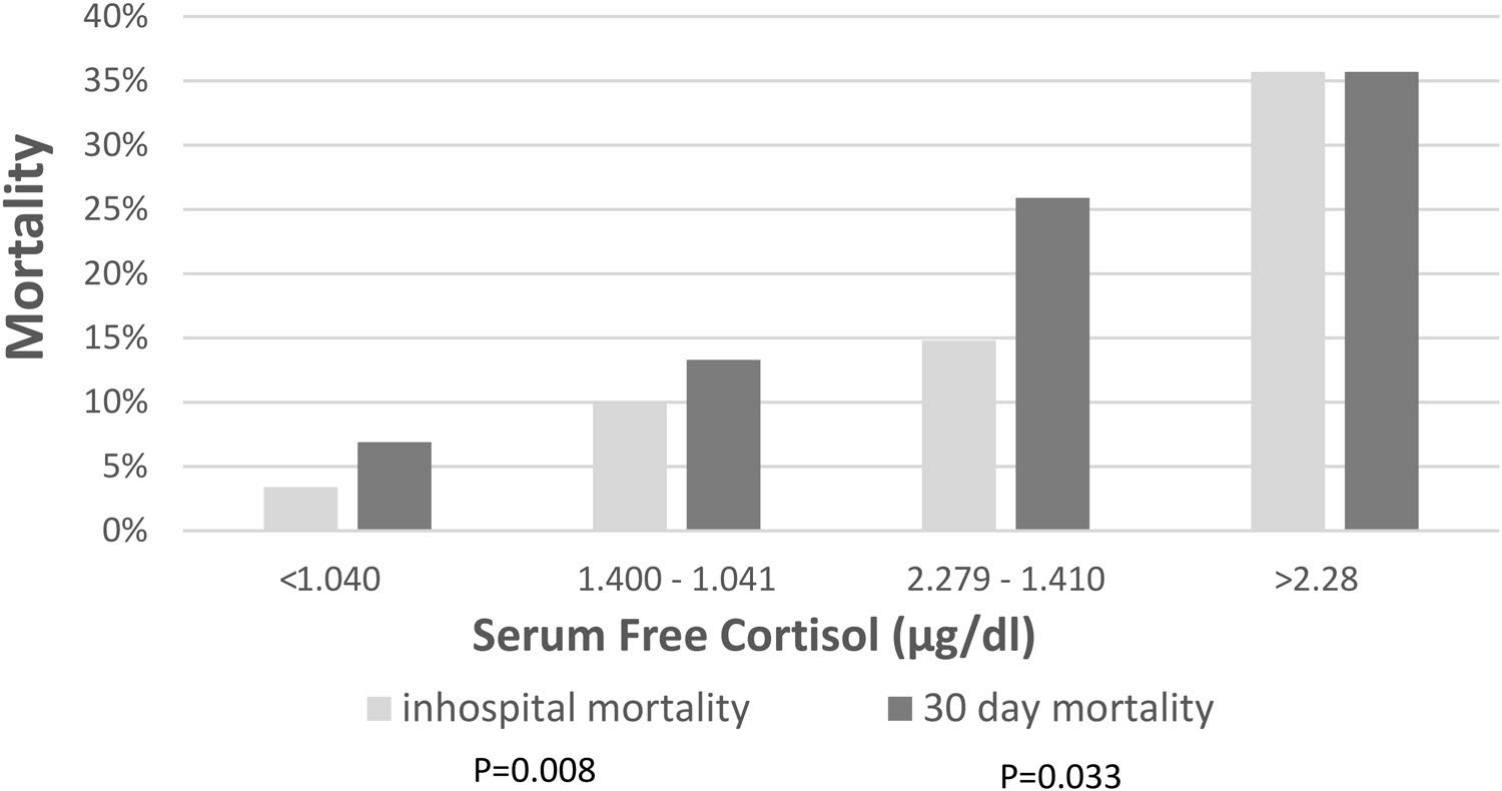
In-hospital (*p* = 0.008) and 30-day (*p* = 0.033) mortality rates are progressively higher with increasing serum free cortisol concentration quartiles in the dexamethasone treated group

SFC concentrations were higher in patients who received dexamethasone [1.4 µg/dl (1.04–2.290] compared to those who did not [1.15 µg/dl (0.77–1.61), *p* < 0.001], reflecting the severity of their disease. Among DT-treated patients, SFC and TC were higher in those who died in hospital or within 30 days, compared with those who survived (Supplementary Table 3). The in-hospital and 30-day mortality rates of DT-treated patients were further analyzed according to SFC quartiles (Fig. 2), indicating that higher SFC concentrations at baseline were associated with increased mortality despite DT treatment. Among 86 patients with severe disease, 81 received DT, of whom 65 survived. Among survivors, 92.3% had TC > 14.6 µg/dl and 90.7% had SFC > 0.73 µg/dl.

### Associations between serum free cortisol and COVID-19 severity

**Fig. 3:**
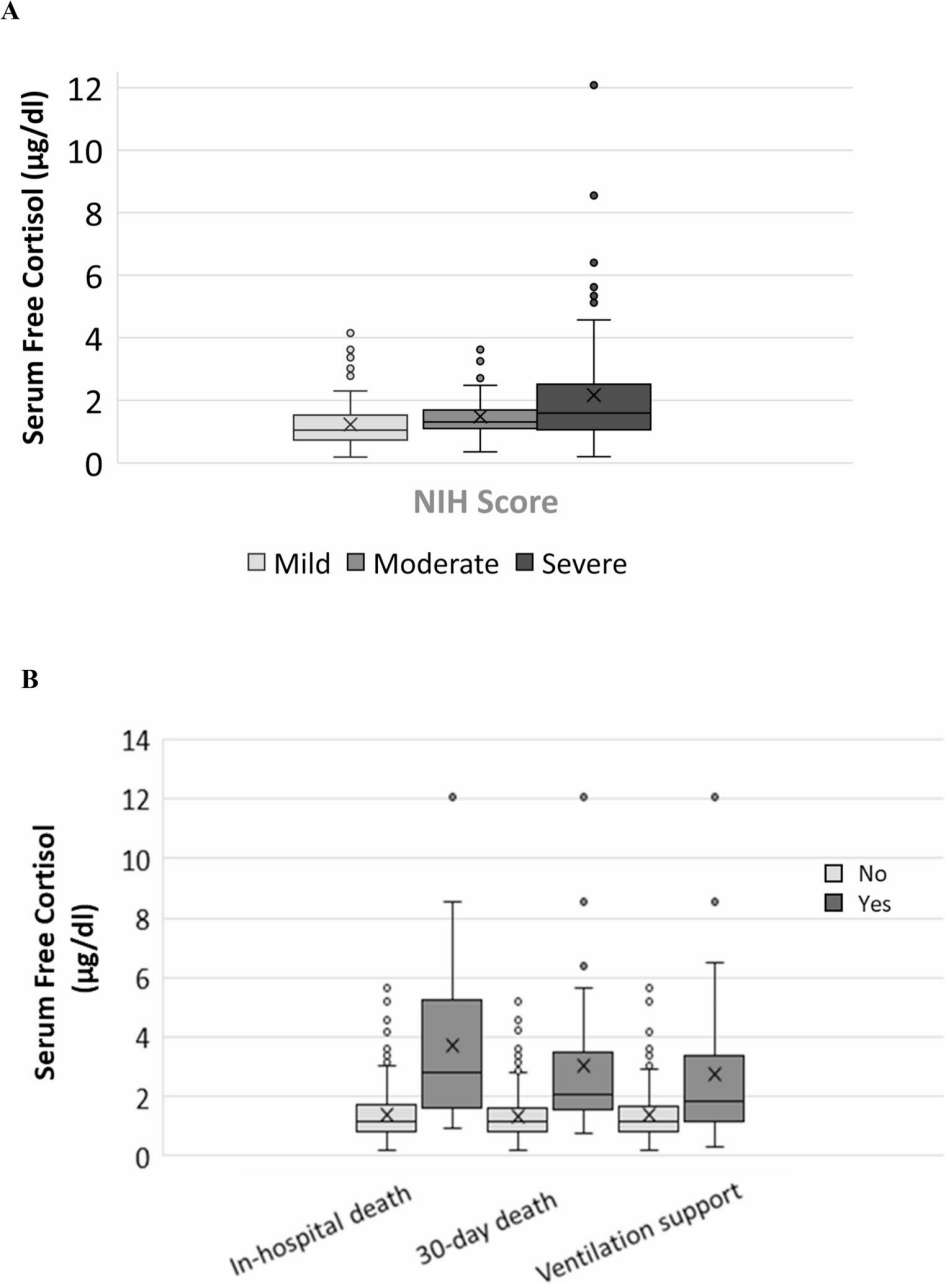
**A** Serum free cortisol is higher in patients presenting with ascending degrees of disease severity appraised by the NIH score, *p* < 0.001. **B** Serum free cortisol levels are higher in patients with adverse clinical outcomes. *P < 0.001 for all pair comparisons

SFC and TC concentrations were higher among patients with comorbidities (Supplementary Table 4) and in those with severe and moderate compared with mild disease (Table 2; Fig. 3A). The percentage of free cortisol increased with disease severity, with higher percentages in severe compared with moderate and mild disease (Table 2).

**Table 2:** Increasing levels of total, free and percentage of free cortisol in parallel to increased COVID-19 severity

NIH score	Mild (*n* = 113)	Moderate (*n* = 42)	Severe (*n* = 86)	*P* value
**Total Cortisol** (IQR), µg/dl	23.0 (16.0-29.3)	28.3 (23.8–35.5)	31.1 (23.5–43.5)	Mild vs. moderate < 0.001 Mild vs. severe < 0.001
**Free Cortisol** (IQR), µg/dl	1.05 (0.73–1.53)	1.31 (1.10–1.69)	1.60 (1.06–2.52)	Mild vs. moderate *p* = 0.008 Mild vs. severe *p* < 0.001
**Free cortisol** (IQR), %	4.85 (4.22–5.80)	4.73 (4.23–5.49)	5.25 (4.38–6.46)	Mild vs. severe *p* = 0.04 Moderate vs. severe *p* = 0.033

A significant positive correlation was found between SFC and several inflammatory markers including IL-6, LDH, ferritin, D-Dimer, fibrinogen, CRP, and neutrophils to lymphocytes ratio (NLR) (*P* < 0.001 for the correlation between each pair of continuous variables (Supplementary Table 5). The strongest correlation was with IL-6, with a Spearman’s R of 0.558, *p* < 0.001 (Supplementary Table 5).

### Associations between serum free cortisol and clinical outcomes

The median hospitalization duration was 5.5 (1-7) days. Thirty-six ventilatory support (14.9%), 21 in-hospital death (8.7%) and 41 30-day death (17%) events were recorded during follow-up. Higher SFC levels (Fig. 3B; Table 3), were associated with in-hospital mortality, 30-day mortality and the need for assisted ventilation (*p* < 0.0001 for all outcomes, Table 3).

**Table 3:** Association between serum total and free cortisol and primary outcomes

	In-hospital mortality		30-day mortality		Ventilation support	
	Survived	Deceased	Survived	Deceased	No	Yes
**Number (%)**	220 (91.3)	21 (8.7)	200 (83)	41 (17)	206 (85.1)	36 (14.9)
**Total Cortisol (IQR)**,** µg/dl**	25.4 (19.4–32.8)	45.1 (26.8–55.8)	25.2 (19.0-31.7)	33.2 (25.1–49.3)	25.4 (19.1–32.0)	35.0 (24.3–52.4)
P value	< 0.001		< 0.001		< 0.001	
**Free Cortisol (IQR)**,** µg/dl**	1.17 (0.83–1.72)	2.83 (1.64–5.27)	1.15 (0.81–1.61)	2.06 (1.59–3.50)	1.17 (0.83–1.69)	1.86 (1.18–3.35)
P value	*P* < 0.001		*P* < 0.001		*P* < 0.001	
**Free Cortisol (IQR)**,** %**	4.86 (4.23–5.65)	7.47 (5.63–9.71)	4.73 (4.21–5.55)	6.26 (5.37–8.34)	4.85 (4.23–5.66)	5.74 (4.77–8.03)
P value	*P* < 0.001		*P* < 0.001		*P* < 0.001	

IQR- interquartile range.

**Fig. 4:**
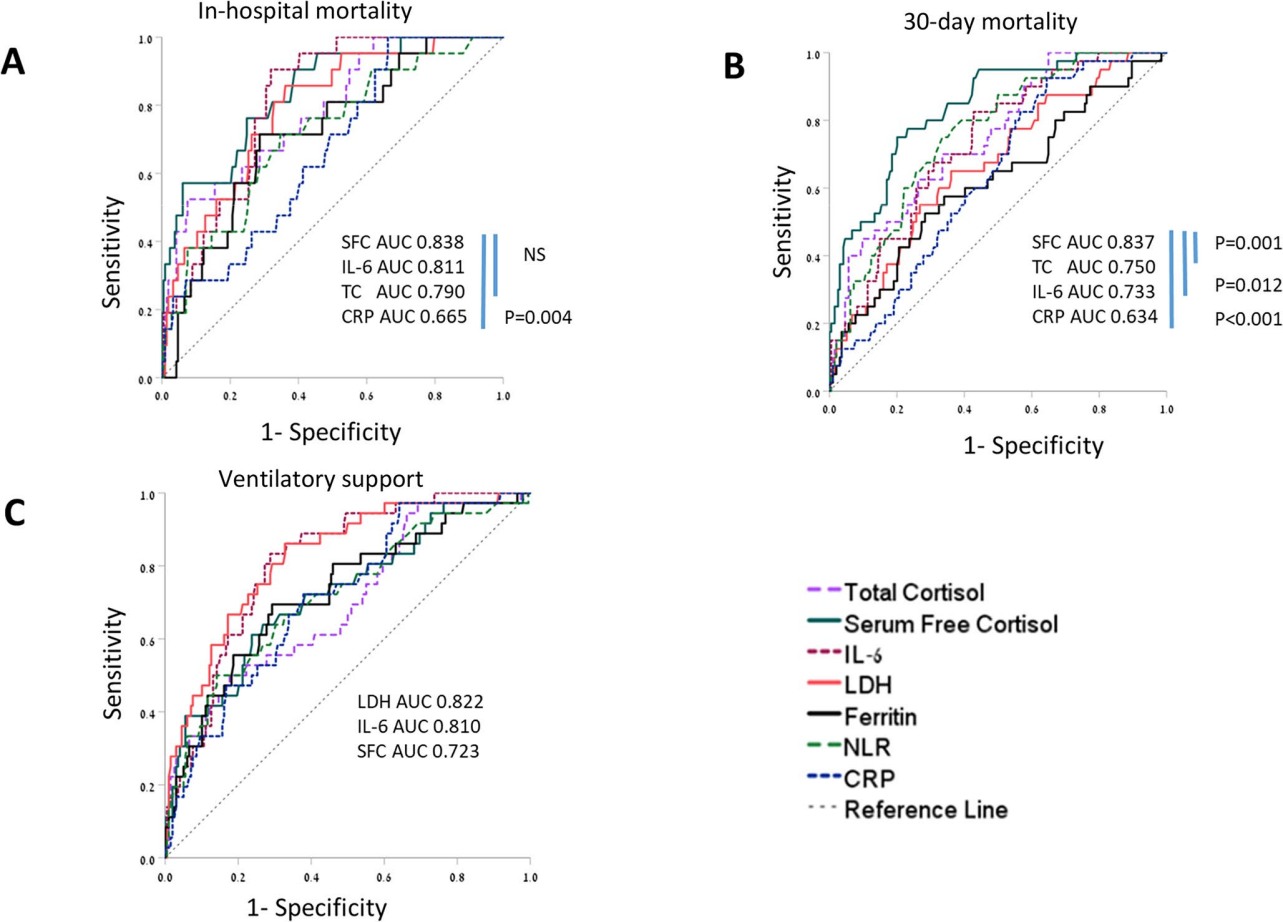
Receiver operating characteristic (ROC)-curves of several biomarkers to predict clinical outcomes. A- ROC of serum free cortisol (SFC, 0.822), Interleukin-6 (IL-6, 0.811) and total cortisol (TC, 0.790) are superior to CRP (0.665, *p* = 0.004) and other inflammatory markers for the prediction of in-hospital mortality; B- ROC of SFC (0.837) is superior to TC (0.750, *p* = 0.001), IL-6 (0.733, *p* = 0.012), CRP (0.634, *p* < 0.001) and other inflammatory markers for the prediction of 30-day mortality, and C- ROC of LDH (0.822), IL-6 (0.810) and SFC (0.723) are similar for the prediction of the need for assisted ventilation

Both in-hospital and 30-day mortality ROC curves demonstrated higher area under the curve (AUC) values for SFC (0.838 and 0.837, respectively) compared with other inflammation biomarkers (Fig. 4A and B). The best cut-off value for SFC was 1.395 for in hospital mortality (sensitivity 0.905, specificity 0.614; Youden’s J 0.518) and 1.730 µg/dl for 30-day mortality (sensitivity 0.732, specificity 0.8; Youden’s J 0.532). In contrast, the AUC curves of LDH (0.822) and IL-6 (0.81) tended to be superior (*p* = 0.072 and *p* = 0.09 respectively) to SFC (0.723) for predicting the need for assisted ventilation, (Fig. 4C).

Multivariate regression analyses were conducted to study the associations between clinical and laboratory variables with the three outcome measures. The initial models included age, chronic kidney disease, hypertension, obesity, dependence in basic activities of daily living (BADL), fever at presentation, desaturation, as well as SFC and IL-6 levels. The final models are presented in Table 4. For each unit increase in SFC the Odds Ratio (OR) for in-hospital mortality, 30-day mortality and the need for assisted ventilation was 1.933 [1.299–2.877], *P* = 0.001; 2.673 [1.77–4.037], *P* < 0.001; and 1.518 [1.136–2.030], *p* = 0.005, respectively.

**Table 4:** Multivariate regression analyses for in hospital mortality, 30-day mortality and need for ventilatory support

Clinical outcome	Confounder	OR (CI)	*P*-value
**In-hospital mortality**	Age	1.068 (1.019–1.120)	0.006
	Serum free cortisol	1.933 (1.299–2.877)	0.001
	Oxygen saturation < 94%	7.127 (1.821–27.887)	0.005
**30-day mortality**	Dependence in BADL	3.57 (1.368–9.315)	0.009
	Age	1.053 (1.016–1.091)	0.005
	Serum free cortisol	2.673 (1.770–4.037)	< 0.001
**Ventilation support**	Oxygen saturation < 94%	9.006 (3.445–23.546)	< 0.001
	Serum free cortisol	1.518 (1.136–2.030)	0.005
Ventilation support *	Obesity	3.647 (1.553–8.562)	0.003
	Serum free cortisol	2.091 (1.531–2.856)	< 0.001

BADL- basic activities of daily living

*- without including desaturation as a variable

## Discussion

In contrast with previous studies that reported central hypoadrenalism in up to 38.5% of hospitalized COVID-19 patients [9, 10, 19], we found that adrenal insufficiency (AI) is a rare occurrence. No patients with mild or moderate disease had morning cortisol levels less than 3–5 µg/dl [22] Furthermore, in patients with mild disease who would not necessarily be expected to mount a significant increase in cortisol secretion, all but 12 had values over 14.6 µg/dl, indicating a normal adrenal reserve based on stimulation test thresholds. In patients with severe disease, only five had values indicating an insufficient response of TC to stress, while six had SFC levels below the expected threshold. Notably, only three patients had TC below 10 µg/dl, the threshold used to diagnose CIRCI. As such, the incidence of AI in patients with severe disease could be between 3.5 and 7%.

Different definitions of AI in patients with diverse degrees of disease severity may explain the discrepancy of reported AI from different groups. Alzahani et al. reported AI in nine of 28 (32%) hospitalized COVID-19 patients [10]. They defined AI as TC < 10.8 µg/dl, but eight of nine of these patients were either asymptomatic or had mild disease. This threshold is significantly higher than the proposed values [22, 23] for subjects in their baseline status, thus probably leading to false positives [10]. Das et al. reported a 38.5% incidence of AI in patients with moderate to severe COVID-19, based on total cortisol (TC) levels lower than 15 µg/dL [9]. The length of illness prior to hospitalization, which was not reported in the publication, could potentially lead to the development of CIRCI if prolonged. In such cases, a cut-off of 10 µg/dL might have been more appropriate. While SFC measurement via equilibrium dialysis is more resource-intensive than immunoassays for total cortisol, it may offer superior clinical value in specific scenarios—particularly in critically ill patients where binding protein alterations limit the utility of total cortisol. We believe that its main clinical importance lies in the diagnosis of adrenal insufficiency in critical care settings.

Our study provides previously unreported information regarding the cortisol secretion status of patients treated with dexamethasone. Our results do not support the hypothesis that dexamethasone’s beneficial effect could be partly mediated by correcting possible hypoadrenalism, at least in most patients. This conclusion is based on the observation that SFC levels in over 90% of dexamethasone-treated patients who survived severe disease were above the cut-off values for a normal response to ACTH stimulation [21].

In accordance to most published studies, we found that patients with severe COVID-19 had markedly higher TC and SFC levels compared to those with mild disease. Furthermore, SFC had a higher AUC to predict in-hospital and 30-day mortality, was more discriminatory of patients with comorbidities and supplanted TC in the multivariate analysis. Our results strongly suggest that SFC is not only a robust indicator of disease severity but also a superior predictor of both in-hospital and 30-day mortality compared to traditional inflammatory markers such as IL-6 and CRP.

Our study is notable for being the first, to our knowledge, to investigate SFC as a distinct prognostic marker in COVID-19. The significance of SFC lies in its bioactive status; free cortisol, unbound to carrier proteins, more accurately reflects the body’s physiological response to stress and infection compared to TC. This biologically active form of cortisol penetrates tissues and exerts its effects at the cellular level, providing a more precise assessment of the systemic impact of severe infections like COVID-19. In contrast, previous small-scale studies focusing on TC yielded conflicting results, highlighting the limitations of TC measurements in the context of acute illness.

Remarkably, our study highlights SFC’s potential to outperform other biomarkers in predicting negative outcomes in COVID-19, which is further supported by parallel findings in other acute illnesses, such as community-acquired pneumonia, where SFC was similarly found to surpass CRP and procalcitonin in prognostic accuracy [27]. Still, we do not propose using SFC as a standalone prognostic marker, but rather in combination with other biomarkers and clinical indicators of disease severity,

Our study has several strengths that enhance the validity of its results. The relatively large size of our cohort and the extensive range of clinical measures collected contribute to the robustness of our analyses, providing a solid foundation for our conclusions. Additionally, patients were systematically stratified based on disease severity, using standardized, readily available clinical instruments, thus allowing for a precise analysis and interpretation of the role of SFC according to patients’ clinical context.

Several limitations may affect the interpretation of our results. Blood samples were initially drawn for the measurement of IL-6, rather than cortisol. This aspect of sample collection may introduce biases related to the timing and conditions under which samples were obtained, which may not coincide with the peak levels of cortisol that are influenced by both diurnal variation and the progression of the illness. Notwithstanding, the diurnal variation is blunted during critical illness, which will elicit high cortisol levels also outside the time period in which cortisol is expected to be higher. Supporting this assumption, there was no difference between TC and SFC levels in samples drawn between 7–10:30 am and those drawn outside this time-period. The absence of ACTH data limits our ability to fully explore the dynamics of the HPA axis. This was due the retrospective design of the study, where the primary focus at the time of blood draw was patient care and the pragmatic constraints of clinical settings during acute COVID-19 management, rather than research hypotheses about the HPA axis. Furthermore, because the patient inclusion depended on the availability of frozen samples, a large number of cases were excluded, raising the possibility that the cohort may not be representative of the overall patient population. The gender imbalance in our cohort, with a significantly higher rate of female patients, constitutes another limitation that necessitates careful interpretation of our results. The high exclusion rate of male subjects exposed to dexamethasone prior to blood sampling may have affected the representation of more severe cases and outcomes within the male subgroup. Finally, the highly elevated serum cortisol levels in the patients with severe disease are in the range observed in patients with Cushing syndrome. None of the patients had documented signs of Cushing syndrome, and there was no clinical indication for targeted evaluation during hospitalization. Nevertheless, we acknowledge the remote possibility that undiagnosed hypercortisolism may have contributed to elevated baseline cortisol levels in rare patients.

In conclusion, adrenal insufficiency is a rare occurrence in hospitalized patients with COVID-19, and the salutary effects of dexamethasone in patients with severe disease is mostly unrelated to adrenal function status. SFC is the strongest predictor of in-hospital and 30-day mortality, emerging as a superior and more sensitive biomarker when compared to other inflammatory parameters in COVID-19. Although COVID-19, as a major lethal threat, has subsided, these findings might be applicable to other, emerging infectious and not infectious processes that are associated with a high rate of negative outcomes.

NIH- National Institutes of Health; IQR- interquartile range.

## Data Availability

Some or all datasets generated during and/or analyzed during the current study are not publicly available but are available from the corresponding author on reasonable request.
